# Long-Term Safety and Efficacy Data of Golodirsen in Ambulatory Patients with Duchenne Muscular Dystrophy Amenable to Exon 53 Skipping: A First-in-human, Multicenter, Two-Part, Open-Label, Phase 1/2 Trial

**DOI:** 10.1089/nat.2021.0043

**Published:** 2022-01-31

**Authors:** Laurent Servais, Eugenio Mercuri, Volker Straub, Michela Guglieri, Andreea M. Seferian, Mariacristina Scoto, Daniela Leone, Erica Koenig, Navid Khan, Ashish Dugar, Xiaodong Wang, Baoguang Han, Dan Wang, Francesco Muntoni

**Affiliations:** ^1^ I-Motion Institute, Hôpital Armand Trousseau, Paris, France.; ^2^ Division of Child Neurology, Centre de Références des Maladies Neuromusculaires, Department of Pediatrics, University Hospital Liège & University of Liège, Liège, Belgium.; ^3^ MDUK Oxford Neuromuscular Centre, University of Oxford, Oxford, United Kingdom.; ^4^ Pediatric Neurology Unit, Università Cattolica del Sacro Cuore Roma, Rome, Italy.; ^5^ Nemo Clinical Centre, Fondazione Policlinico Universitario A Gemelli IRCCS, Rome, Italy.; ^6^ John Walton Muscular Dystrophy Research Centre, Newcastle University and Newcastle Hospitals NHS Foundation Trust, Newcastle upon Tyne, United Kingdom.; ^7^ Dubowitz Neuromuscular Centre, University College London, Great Ormond Street Institute of Child Health, London, United Kingdom.; ^8^ National Institute for Health Research Great Ormond Street Hospital Biomedical Research Centre, London, United Kingdom.; ^9^ Sarepta Therapeutics, Inc., Cambridge, Massachusetts, USA.

**Keywords:** golodirsen, Duchenne muscular dystrophy, exon skipping

## Abstract

The aim of this Phase 1/2, 2-part, multicenter trial was to report clinical safety and efficacy of long-term golodirsen treatment among ambulatory patients with exon 53 skip-amenable Duchenne muscular dystrophy (DMD). Part 1 was a 12-week, randomized, double-blind, placebo-controlled, dose-titration study followed by 9-week safety review. Part 2 was a 168-week, open-label evaluation of golodirsen 30 mg/kg. Part 1 primary endpoint was safety. Part 2 primary endpoints were dystrophin protein expression and 6-minute walk test (6MWT); secondary endpoints were percent predicted forced vital capacity (FVC%p) and safety. *Post hoc* ambulation analyses used mutation-matched external natural history controls. All patients from Part 1 (golodirsen, *n* = 8; placebo, *n* = 4) plus 13 additional patients entered Part 2; 23 completed the study. Adverse events were generally mild, nonserious, and unrelated to golodirsen, with no safety-related discontinuations or deaths. Golodirsen increased dystrophin protein (16.0-fold; *P* < 0.001) and exon skipping (28.9-fold; *P* < 0.001). At 3 years, 6MWT change from baseline was −99.0 m for golodirsen-treated patients versus −181.4 m for external controls (*P* = 0.067), and loss of ambulation occurred in 9% versus 26% (*P* = 0.21). FVC%p declined 8.4% over 3 years in golodirsen-treated patients, comparing favorably with literature-reported rates. This study provides evidence for golodirsen biologic activity and long-term safety in a declining DMD population and suggests functional benefit versus external controls. Clinical Trial Registration number: NCT02310906.

## Introduction

Duchenne muscular dystrophy (DMD) is an X-linked, recessive, degenerative neuromuscular disease caused by mutations in the dystrophin (*DMD*) gene that disrupt the messenger RNA (mRNA) open reading frame, preventing translation of the dystrophin protein [1,2]. DMD is the most frequent hereditary muscle disease and affects 1 in every 3,500 − 5,000 boys born worldwide [3,4]. It is characterized by progressive muscle wasting and is universally fatal, with mean age of survival around the late 20s [5–7]. When treated with corticosteroids, patients <7 years of age typically improve their 6-minute walking test (6MWT) distance and may improve their North Star Ambulatory Assessment (NSAA) scores, but patients >7 years of age tend to exhibit progressive deterioration and declining ambulatory function, with loss of ambulation ∼13 years of age [8–11].

Although progressive functional decline is common to all patients with DMD, natural history studies have revealed differences in the disease trajectories among patients according to steroid treatment, genetic modifiers, and, importantly, different underlying *DMD* mutations that have implications for their therapeutic management [1,8,12,13]. Mutations amenable to exon 53 skipping are present in ∼8% of patients with DMD, and multiple independent studies have confirmed that these patients have more severe phenotypes compared with other patients with DMD, including earlier onset of decline, poorer muscle strength and function, and earlier loss of ambulation [1,8,14,15].

Exon-skipping therapies are designed to restore the *DMD* open reading frame and enable translation of internally shortened but functional dystrophin proteins [16,17]. Golodirsen [18] is one of four approved DMD-targeted exon-skipping therapies (the others being eteplirsen [19], viltolarsen [20], and casimersen [21]). Golodirsen is a phosphorodiamidate morpholino oligomer (PMO) designed for sequence-specific antisense binding to *DMD* pre-mRNA to induce skipping of exon 53. It was approved by the U.S. Food and Drug Administration in 2019 for the treatment of DMD in patients who have a confirmed mutation amenable to exon 53 skipping [18]. Approval was based on an observed increase in dystrophin protein expression after treatment.

This first-in-human study of golodirsen aimed to assess its long-term safety and biologic and clinical efficacy in a population of patients with DMD amenable to exon 53 skipping who were at an age associated with progressive deterioration and declining ambulatory function. Here, we report the results of the long-term, open-label part of the study, which evaluated golodirsen safety for up to 189 weeks and efficacy over 144 weeks.

## Materials and Methods

### Study design

This Phase 1/2, multicenter, 2-part study (NCT02310906) enrolled patients at four centers in France, Italy, and the United Kingdom [8]. Part 1 was a randomized, double-blind, placebo-controlled, dose-titration study to assess the safety, tolerability, and pharmacokinetics of four escalating doses of intravenous (IV) golodirsen over 12 weeks, followed by a 9-week safety review [22]. Part 2 was a long-term, 168-week, open-label evaluation of the biologic efficacy (at week 48), clinical efficacy (at week 144), and safety of golodirsen IV 30 mg/kg in patients with DMD amenable to exon 53 skipping. Clinical Trial Registration number is NCT02310906.

The Institutional Review Board or Independent Ethics Committee at each individual site reviewed and approved the protocol and consent forms. Written informed consent from each patient's parent(s) or legal guardian(s) and written assent from each patient were obtained. The study was designed and monitored in accordance with the ethical principles of the International Conference on Harmonisation Good Clinical Practice as required by the major regulatory authorities, and in accordance with the Declaration of Helsinki.

### Patients

Eligible patients were male, 6–15 years old, with an established clinical diagnosis of DMD and a confirmed genetic mutation amenable to exon 53 skipping (except for untreated patients enrolled in Part 2, whose mutations were not amenable to exon 53 skipping). Participants were required to have stable cardiac and pulmonary function, and to be on a stable dose (or dose equivalent) of oral corticosteroids for ≥24 weeks before study initiation. Functional criteria included an ability to walk ≥250 m on the 6MWT at both screening and baseline and achieve a rise time <7 s (Gowers's) or an NSAA total score >17.

Key exclusion criteria were the use of any pharmacologic treatment, aside from corticosteroids, that may have affected muscle strength or function within 12 weeks of study entry; current or previous treatment with experimental agents including BMN-195, PRO053, or other experimental treatments within 12 weeks; left ventricular ejection fraction <50% or corrected QT interval >450 ms; or percent predicted forced vital capacity (FVC%p) <50% at screening/baseline or need for nocturnal ventilation.

### Treatment cohorts

**FIG. 1.**
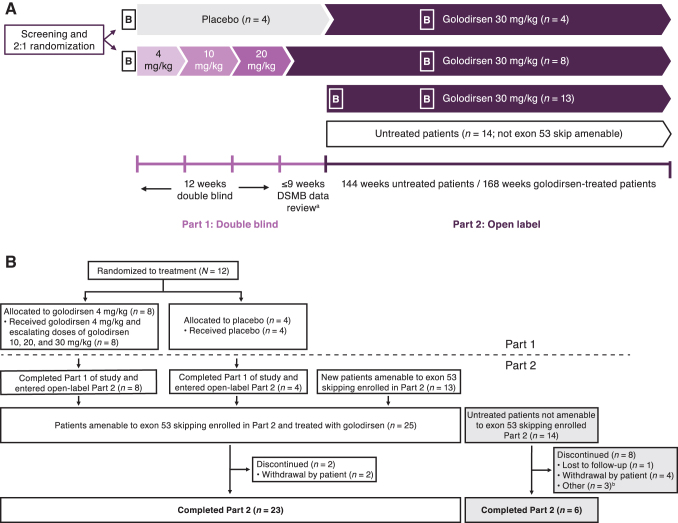
**(A)** Study design. Part 1 was a double-blind, placebo-controlled, dose-titration period; each dose level was administered for ≥2 weeks. Part 2 was an open-label extension period, including all patients from Part 1 plus 13 new patients amenable to exon 53 skipping. The untreated arm consisted of patients not amenable to exon 53 skipping and, per protocol, was not a control group but was included to evaluate DMD natural history and exploratory biomarkers. Adapted with permission from Frank *et al.* [21]. DOI: https://doi.org/10.1212/WNL.0000000000009233. **(B)** Patient disposition. ^a^Patients continued on treatment as randomized through enrollment and DSMB review. ^b^Reasons included enrollment in a therapeutic study (*n* = 2) and personal reasons (*n* = 1). B, biopsy; DMD, Duchenne muscular dystrophy; DSMB, Data Safety Monitoring Board.

In Part 1, patients were randomized 2:1 to receive either a weekly IV infusion of golodirsen or placebo at escalating dose levels, each for ≥2 weeks: 4 mg/kg in weeks 1 and 2, 10 mg/kg in weeks 3 and 4, 20 mg/kg in weeks 5 and 6, and 30 mg/kg beginning at week 7 (Fig. 1). In Part 2, treated patients from Part 1 and a further cohort of new patients with DMD amenable to exon 53 skipping received golodirsen 30 mg/kg/week for 168 weeks. In addition to these, a cohort of patients with mutations not amenable to exon 53 skipping was recruited to Part 2 of the study to evaluate exploratory biomarkers in patients with DMD with other genotypes (biomarker data to be reported elsewhere) and the natural history of the disease over 144 weeks. For treated patients who participated in Parts 1 and 2, the total study duration was 189 weeks.

### Study endpoints and assessments

The primary objective of Part 1 was to evaluate the safety and tolerability of four escalating dose levels of golodirsen versus placebo. Safety assessments included adverse events (AEs), vital signs, physical examinations, clinical laboratory evaluations, electrocardiograms, and echocardiograms. Investigators assessed the severity of all AEs as mild, moderate, or severe, and determined whether AEs were related/unrelated to study treatment, procedures, and/or underlying disease.

AEs were considered treatment emergent (TEAEs) if they started, worsened, or became serious on or after the start of the first infusion and within 28 days after the last dose of study drug, or before receiving the first dose in the extension study. Serious AEs were defined as death, or events that were life threatening or resulted in inpatient hospitalization, persistent or significant disability/incapacity, or an important medical event. The secondary objective of Part 1 was pharmacokinetics (reported elsewhere [22]).

The primary biologic objective of Part 2 was to compare dystrophin expression in muscle biopsy samples at week 48 with baseline. The primary biologic endpoint was the change from baseline to week 48 in dystrophin protein levels as determined by western blot. The secondary biologic endpoints were change from baseline to week 48 in dystrophin intensity by immunohistochemistry, and exon 53 skipping determined using reverse transcription polymerase chain reaction. Biopsy samples were also examined *post hoc* for fetal/developmental myosin expression, a biomarker of myofiber regeneration that occurs after degeneration and is a prominent feature in DMD progression. Procedures for biologic analyses have been described previously [22–24].

The primary efficacy objective of Part 2 was to assess changes from baseline in ambulation in the treated patients; secondary efficacy objectives included assessment of respiratory function. The primary efficacy endpoint was change from baseline to week 144 on the 6MWT. Patients were considered to have lost ambulation if they received a score of 0 on both the NSAA walk and run components, or if the patient was unable to complete the NSAA test due to being nonambulatory at the time of the assessment. The secondary efficacy endpoint was change from baseline to week 144 in FVC%p. Safety was a secondary objective for Part 2.

Exploratory objectives (to be reported elsewhere) were to assess leg muscle morphology using magnetic resonance imaging, magnetic resonance spectroscopy, serum biomarkers, and extremity function and strength.

### *Post hoc* ambulation analysis

To obtain data on golodirsen functional efficacy, comparisons were conducted with matched exon 53 skip-amenable natural history controls. External control patients were identified from a longitudinal multicenter cohort study in Italy, Belgium, and the United Kingdom [8], and matched to the golodirsen-treated group based on age (≥6 years), current steroid use, 6MWT distance (≥250 m), and ability to rise from floor. Sufficiently matched longitudinal external control data were not available for pulmonary function; therefore, *post hoc* analyses were not possible for FVC%p.

### Statistical analysis

Sample size for this study was based on qualitative considerations; no formal sample size calculations were performed. For Part 1, the safety set included all randomized patients who received ≥1 dose of study drug (golodirsen or placebo). For Part 2, the safety set included all randomized patients from Part 1, all Part 2 patients amenable to exon 53 skipping who received any amount of study drug, and all untreated patients who entered Part 2. The efficacy set comprised of all randomized patients from Part 1 and all Part 2 patients who had ≥1 postbaseline functional assessment.

AEs were analyzed using descriptive statistics. For all analyses, baseline was the last evaluation before golodirsen initiation. Changes in dystrophin expression were analyzed using a one-sample permutation *t*-test. In a *post hoc* analysis, correlation of exon skipping and dystrophin expression was analyzed using Spearman's correlation. *Post hoc* comparison of 6MWT with matched exon 53 skip-amenable natural history external controls was conducted using a two-sample *t*-test; loss of ambulation was compared with controls using Fisher's exact test. No formal comparisons were made for respiratory data. For all hypothesis testing, the two-sided significance level was 0.05 with no formal adjustment for multiplicity.

## Results

### Patients amenable to exon 53 skipping

A total of 12 patients were included in Part 1 (golodirsen, *n* = 8; placebo, *n* = 4). All patients completed Part 1 and continued into Part 2. An additional 13 patients entered the trial at the start of Part 2 (Fig. 1), resulting in a final cohort of 25 patients receiving open-label golodirsen 30 mg/kg/week in Part 2. Patients were 8.4 years of age on average (4 were <7 years of age), with average 35 months of corticosteroid use, an average 6MWT distance of 406 m, and an average FVC%p of 93% (Table 1). The mean duration of time on study during the combined study periods was 170.1 weeks, and patients received a mean of 164 infusions (median 167 infusions). Two patients withdrew before completion of Part 2 (patient decision at 73 and 98 weeks, respectively).

**Table 1.** Baseline Characteristics of Golodirsen-Treated Patients and Matched Exon 53 Skip-Amenable Natural History Controls

Baseline characteristic^a^	Golodirsen-treated patients (*n* = 25)	Matched exon 53 skip-amenable natural history controls (*n* = 19)	*P*
Age, years	8.4 (2.2)	9.1 (1.7)	0.17
Range	6–13	6–11.6	
Height, cm	120.5 (10.1)	N/A	
Weight, kg	28.4 (9.0)	N/A	
BMI, kg/m^2^	19.1 (3.7)	N/A	
Mutation, *n* (%)			
45 − 52	8 (32.0)	2 (10.5)	
48 − 52	5 (20.0)	9 (47.4)	
49 − 52	5 (20.0)	3 (15.8)	
50 − 52	4 (16.0)	1 (5.3)	
52	3 (12.0)	4 (21.1)	
6MWT distance, m	405.8 (55.1)	382.1 (55.9)	0.17
Range	290–512	300–489	
Time to rise from floor, s	5.9 (3.5)	6.2 (3.1)	0.76
Range	2.3–18.6	3–14.9	
NSAA	23.6 (5.0)	N/A	
Range	13–33	N/A	
FVC%p	92.7 (24.0)	N/A	
Range	16.4–137.8	N/A	
Time since DMD diagnosis, months	55.8 (24.8)	N/A	
Range	16.1–122.9		
Duration of corticosteroid use, months	35.3 (24.4)	N/A	
Range	8.9–97.7		
Frequency of corticosteroid administration, *n* (%)			
Continuous	19 (76.0)	7 (36.8)	
Intermittent	6 (24.0)	12 (63.2)	
Corticosteroid type, *n* (%)			
Deflazacort	12 (48.0)	N/A	
Prednisone	13 (52.0)	N/A	

Values are mean (SD) unless noted otherwise.

^a^ For golodirsen-treated patients, baseline was defined as the last assessment before golodirsen initiation.

6MWT, 6-minute walk test; BMI, body mass index; DMD, Duchenne muscular dystrophy; FVC%p, percent predicted forced vital capacity; N/A, not available; NSAA, North Star Ambulatory Assessment; SD, standard deviation.

### Safety

Safety was assessed in all golodirsen-treated patients (*n* = 25), with exposure up to 189 weeks (mean 167 weeks). In both the double-blind period (Part 1) and the open-label period (Part 2), all patients experienced ≥1 AE (Table 2). Most AEs were mild, nonserious, and assessed by the investigator as unrelated to golodirsen. Five AEs were deemed severe (all were events of fracture or inability to walk), but none were considered serious.

**Table 2.** Adverse Events Overview

	Part 1		Combined Parts 1 and 2
AEs,* n *(%)	Placebo (*n* = 4)	Golodirsen (*n* = 8)	Total golodirsen (*n* = 25)
Patients with ≥1 AE, *n* (%)	4 (100)	8 (100)	25 (100)
Related to study drug	2 (50.0)	5 (62.5)	9 (36.0)
Serious	0	0	4 (16.0)
Leading to study drug discontinuation	0	0	0
Total AEs by severity, *n*	23	69	860
Mild	22	68	831
Moderate	1	1	24
Severe	0	0	5

AE, adverse event.

No anaphylaxis or serious hypersensitivity events were reported. Infusions were well tolerated, and the majority of infusion-related reactions were mild and nonserious; none were severe. Of infusion-related reactions, five were possibly related to golodirsen, including pyrexia, rash, tachycardia, erythema, and decreased blood pressure (all mild). There was no evidence of serious kidney toxicity. Two patients experienced mild renal AEs (both proteinuria) that were transient and nonserious, and resolved spontaneously. No patient discontinued treatment because of AEs. There were no deaths in the study.

In total, nine patients experienced treatment-related TEAEs (Table 3). Treatment-related pyrexia, headache, and proteinuria each occurred in >1 patient. Cardiac events possibly related to golodirsen were reported in two patients [tachycardia (*n* = 1; 30 minutes after infusion) and syncope (*n* = 1; 5 days after infusion)]; both were nonserious and mild, and both resolved without intervention and did not lead to treatment discontinuation. Syncope did not recur during the study. Two further instances of heart rate >125 bpm occurred 60 min post infusion in the patient with tachycardia. Four (15%) patients experienced seven serious AEs (vomiting, pyrexia, hypocalcemia, hematemesis, viral gastroenteritis, convulsion, and tonsillar hypertrophy); all were deemed to be unrelated to golodirsen treatment.

**Table 3.** Study Drug-Related Treatment-Emergent Adverse Events in Combined Parts 1 and 2

AEs,* n *(%)	Total golodirsen group (*n* = 25)
Any TEAE related to study drug	9 (36.0)
Pyrexia	3 (12.0)
Headache	2 (8.0)
Proteinuria	2 (8.0)
Syncope	1 (4.0)
Erythema	1 (4.0)
Rash	1 (4.0)
Skin exfoliation	1 (4.0)
Sinus tachycardia	1 (4.0)
Tachycardia	1 (4.0)
Gastroenteritis	1 (4.0)
Blood pressure decreased	1 (4.0)
Hyperglycemia	1 (4.0)

TEAE, treatment-emergent adverse event.

To deliver golodirsen, a port-a-cath was inserted in six patients before starting study drug, and in one patient after the first three doses of golodirsen. The total number of infusions given through port was 1,185, accounting for 94% of expected doses in those patients (6% of doses missed). Median age of patients receiving a port was 10.7 years. AEs related to the port were reported in five patients, and included catheter site bruising (*n* = 3), catheter site pain (*n* = 3), catheter site rash (*n* = 1), peripheral swelling (*n* = 1), and infection related to the port (*n* = 1). None of the patients who received a port withdrew from the study.

### Exon skipping and dystrophin expression

**FIG. 2.**
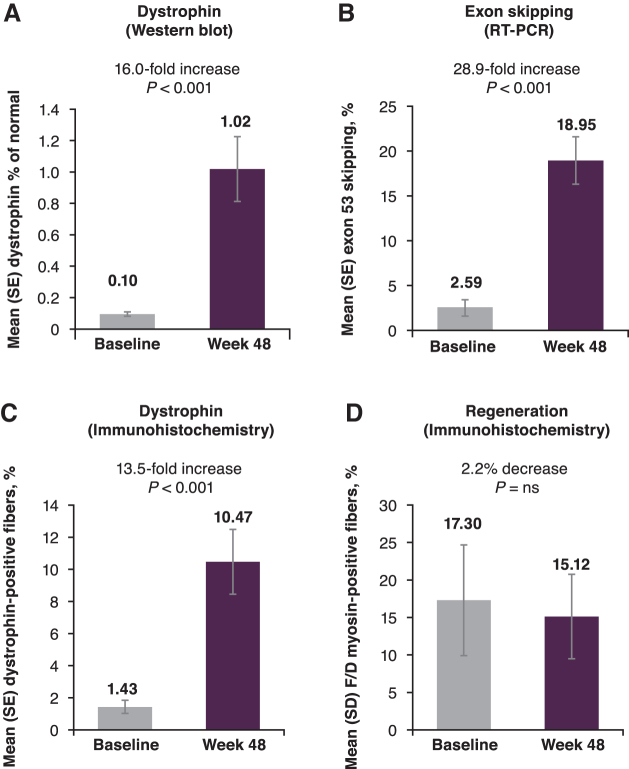
**(A)** Mean dystrophin protein by western blot, **(B)** exon skipping by RT-PCR, **(C)** dystrophin protein by immunohistochemistry, and **(D)** percentage of F/D myosin-positive fibers were measured at baseline and after 48 weeks of golodirsen treatment. F/D, fetal/developmental; ns, not significant; RT-PCR, reverse transcription polymerase chain reaction.

We have previously reported that at week 48, exon skipping and dystrophin expression were both significantly increased (all *P* < 0.001) among patients treated with golodirsen, and positive correlation was observed between exon 53 skipping and dystrophin production (Spearman's correlation coefficient: 0.50; *P* < 0.02) [22]. Treatment with golodirsen (Parts 1 and 2 combined) resulted in a significant, 16.0-fold mean increase in dystrophin protein levels detected by western blot, from a baseline mean of 0.095% of normal levels, to 1.019% of normal at week 48 (*P* < 0.001; Fig. 2A).

Similarly, the level of exon 53-skipped *DMD* gene expression was found to be increased by 28.9-fold (*P* < 0.001; Fig. 2B). We also reported that the percentage of dystrophin-positive fibers was significantly increased at week 48 (13.5-fold increase; *P* < 0.001; Fig. 2C) [22], and that myofiber regeneration decreased after golodirsen treatment, indicated by fewer fibers positive for fetal/developmental myosin at week 48 compared with baseline (Fig. 2D) [23].

### Ambulatory and pulmonary function

Mean 6MWT distance at baseline was 405.8 m for golodirsen-treated patients and declined by 26.1, 64.6, and 99.0 m at weeks 48, 96, and 144, respectively (Table 4). Two of 25 patients lost ambulation. Among golodirsen-treated patients, FVC%p declined by 8.4% over 3 years of treatment, from a mean FVC%p of 92.7% at baseline to 83.8% at week 144.

**Table 4.** Ambulatory and Pulmonary Function in Golodirsen-Treated Patients

Endpoints	Baseline^a^	Week 48	Week 96	Week 144
6MWT, m	*n* = 25	*n* = 23	*n* = 24	*n* = 22
Mean (SD)	405.8 (55.1)	378.9 (93.2)	344.1 (128.9)	311.0 (143.4)
Range	290 − 512	81 − 541	0 − 523	0 − 481
Mean (SD) change from baseline		−26.1 (65.1)	−64.6 (105.1)	−99.0 (123.8)
Loss of ambulation	*n* = 25	*n* = 25	*n* = 24	*n* = 23
*n* (%)	0	0	1 (4.0)	2 (9.0)
FVC%p	*n* = 25	*n* = 24	*n* = 23	*n* = 23
Mean (SD)	92.7 (24.0)	92.5 (18.7)	93.9 (17.6)	83.8 (23.2)
Range	16.4–137.8	41.9–129.9	37.4–124.9	7.8–121.1
Mean (SD) change from baseline		−0.63 (21.5)	0.79 (23.8)	−8.38 (29.5)

^a^ Baseline FVC%p for placebo patients was defined as Part 2 baseline FVC%p, and inclusion criteria (FVC%p > 50%) for screening were not applied at that time.

### *Post hoc* ambulation analysis

From the external control natural history cohort [8], 28 patients were identified who were amenable to exon 53 skipping and had longitudinal 6MWT assessments for comparison. Of these, 19 patients were successfully matched according to the golodirsen-treated group inclusion criteria; 9 patients did not meet matching criteria and were excluded (age <6 years, *n* = 6; steroid naive, *n* = 1; unable to rise, *n* = 2). Patients treated with golodirsen had a numerically longer mean baseline 6MWT distance compared with the matched controls, but this was nonsignificant (*P* = 0.17); otherwise, available baseline characteristics were similar between these two groups (Table 1).

**FIG. 3.**
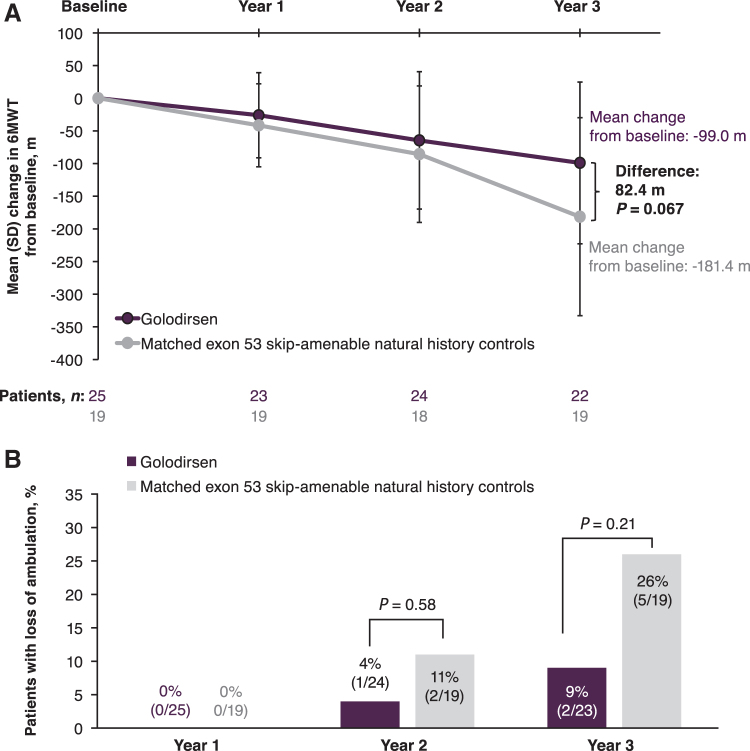
Ambulatory function: **(A)** 6MWT distance and **(B)** loss of ambulation over 3 years in golodirsen-treated patients and matched exon 53 skip-amenable natural history external controls. 6MWT, 6-minute walk test.

Control patients' 6MWT distance declined by a mean of 181.4 m [standard deviation (SD), 151.6; range, −401 to 56] after 3 years compared with baseline. In contrast, golodirsen-treated patients maintained a more stable trajectory, with a mean decline from baseline of 99.0 m (SD, 123.8; range, −368 to 144) after 3 years (*P* = 0.067 between groups; Fig. 3A). This difference emerged over time, as no difference between treated and untreated patients was observed at the time points before year 3. Among natural history controls, 5 of 19 patients (26%) had lost ambulation over 3 years, compared with 2 of 25 (9%) of those who received golodirsen (*P* = 0.21; Fig. 3B).

### Untreated arm (not amenable to exon 53 skipping)

Genotypes of patients in the untreated arm are presented in Table 5, and included genotypes associated with a milder clinical course, such as mutations amenable to exon 44 skipping. The untreated arm was intended to evaluate the natural history of disease and, specifically, exploratory biomarkers that are still under investigation. Per protocol, it was not considered a control group for efficacy comparison of golodirsen-treated patients, given the differences in disease trajectories demonstrated for patients with differing DMD genotypes [1,8,12,13].

**Table 5.** Untreated Arm Functional Outcomes

Endpoints	Baseline	Week 144
6MWT distance	*n* = 13	*n* = 6
Mean (SD)	455.1 (51.1)	278.7 (188.9)
Range	351–539	0–525
FVC%p	*n* = 13	*n* = 5
Mean (SD)	97.9 (18.3)	77.5 (18.6)
Range	60.85–120.51	53.86–99.96

Genotypes in the untreated arm included (*n* = 1 each) 1–47, 3–7, 7–17, 8–43, 10–21, 22–25, 30–43, 35–43, 45, 45–50, 46–51, 46–52, 51, and 61–62.

The untreated arm in Part 2 included 14 patients. Eight patients discontinued before the end of the follow-up period (one was lost to follow-up, four withdrew, two enrolled in a therapeutic study, and one discontinued for personal reasons). Mean (SD) age was 8.5 (1.9) years (range, 6 − 12 years). Outcomes for the untreated patients are shown in Table 5. Declines in both ambulatory and pulmonary functions were observed from baseline to week 144.

## Discussion

The results presented herein provide evidence for the biologic activity and long-term safety of golodirsen in a declining DMD population, supporting evaluation of golodirsen in an ongoing Phase 3 trial (NCT02500381). Although the study was not powered or designed to demonstrate efficacy, *post hoc* analysis using an external control suggests possible functional benefit after a 3-year exposure.

The data show that long-term treatment with golodirsen, assessed at a dose of 30 mg/kg/week, is well tolerated in patients with DMD amenable to exon 53 skipping. During long-term treatment, AEs were generally mild, nonserious, and unrelated to golodirsen, and there were no discontinuations due to safety. Most AEs were consistent with conditions that may be anticipated in the pediatric population, and with complications or comorbidities of the underlying DMD.

There was no suggestion of a serious risk of kidney abnormality or toxicity. Mild cardiac events possibly related to golodirsen were reported in two patients (tachycardia in an 8-year-old patient on day 8 and syncope in a 13-year-old patient on day 13), but both were nonserious, resolved, and did not lead to treatment discontinuation. These cardiac events may have been confounded by underlying disease. No anaphylaxis or serious hypersensitivity was observed. 

The safety profile of golodirsen is consistent with that of other approved PMOs targeting different exons, such as eteplirsen and casimersen [19,21,22,25–27]. Both of these have been previously shown to increase dystrophin production, with a tolerability profile revealing neither renal nor hepatic safety signals, and serum chemistry and properties within expectations given the progression of DMD.

Seven of 25 patients in this study received a venous port for administration of golodirsen to ease the burden of weekly IV infusions. In these patients, use of the port to receive golodirsen was successful, but did not affect the level of treatment compliance (94% in patients with a port vs. 95% in patients without). AEs related to the port were generally limited to pain and bruising; infection related to the port was observed in only one patient (out of seven who had a port). All patients who received ports completed the study, and no withdrawals were due to port-related AEs. The successful use of ports eases the administration burden for golodirsen and the distress for patients associated with weekly cannulation.

The potential functional benefits of golodirsen emerged with detection of increased exon skipping and dystrophin protein in golodirsen-treated patients, and these two measures of biologic activity were positively correlated. Over 48 weeks, exon skipping increased 28.9-fold, dystrophin protein increased 16.0-fold, reaching ∼1%, and percentage of dystrophin-positive fibers increased 13.5-fold compared with baseline [22].

These complementary bioanalytical techniques were used to confirm that the relative increase in dystrophin production was correlated with correct localization and distribution of the protein at the sarcolemma of the muscle fibers. Animal studies [28] and clinical studies [29,30] have shown that even low levels of dystrophin can improve functional outcomes in DMD and are associated with milder dystrophinopathy. In one recent study, achieving dystrophin quantities <0.5% of normal was associated with a milder clinical phenotype and a delay to loss of ambulation, suggesting that any increase in dystrophin protein is beneficial [30]. Further, studies of other PMOs show that dystrophin accumulates with long-term treatment, including at time points beyond 48 weeks [26,31,32].

*In vitro* studies have also demonstrated the molecular functionality of dystrophin protein produced by DMD myotubes after golodirsen treatment [24]. Consistently, we have previously demonstrated in post-treatment biopsy data from golodirsen-treated patients that increased dystrophin was associated with a 2.2% decrease in fibers positive for the regeneration marker fetal/developmental myosin [23].

Although this change did not reach statistical significance, a previous trial on an unsuccessful DMD treatment showed a 1.2% increase [confidence interval (95% CI) −1.1 to 3.4] in myofibers positive for fetal/developmental myosin over 48 weeks [33]. Together, these results suggest a histologic benefit with golodirsen treatment and provide evidence of protection from the ongoing muscle dystrophic process. In aggregate, the biologic data from our study demonstrate target engagement by golodirsen, and achievement of dystrophin levels and histologic benefits that are likely to predict clinical benefit.

Comparative efficacy assessments in DMD therapy trials are complicated by the small numbers of patients with matched genetic mutations and the unsuitability of patients with nonmatched mutations as controls. This is particularly relevant for early (Phase 1/2) studies, in which a placebo arm is usually not planned for longer term observation. Although longer term placebo-controlled studies are desirable to increase the level of evidence, there is limited participant willingness to be exposed only to placebo during a time of irreversible function loss [34]. For exon-skipping therapies, maximal therapeutic effects may not be apparent in short-term studies [35]. Studies of other exon-skipping therapies have also met with these challenges in study design, and have used mutation-matched external controls to compare efficacy assessments [36,37].

Therefore, an external natural history cohort, matched for mutation class and baseline functional capability, offered the most relevant comparator group to assess golodirsen efficacy [8]. Compared with these matched exon 53 skip-amenable natural history controls, golodirsen treatment possibly attenuated ambulatory function loss, slowing decline and helping treated patients maintain a more stable trajectory. Similarly, a smaller proportion of golodirsen patients lost ambulation over the 3-year study period than natural history patients (9% vs. 26%).

Although statistical significance could not be reached with these small sample sizes, this unpowered study suggests that golodirsen treatment elicits a promising departure from natural history. The gradual divergence of the evolution in comparison with untreated patients is consistent with the mode of action and the accumulation of dystrophin with continued treatment.

Patients selected for the study were in an age range (6 − 15 years) in which the DMD natural history is in a phase of progressive deterioration, including declining ambulatory function leading to loss of ambulation [8–11]. The study also included four patients <7 years of age; patients at the younger end of the age range may have shown functional improvement due to the effect of physiologic growth and development, confounding assessment of treatment effect. Nevertheless, these patients followed a trajectory consistent with the overall study population.

Numerous publications have documented rates of FVC%p decline in mixed-genotype cohorts, with an average annual rate of loss between 4.5% and 7% [12,38–47], including patients taking corticosteroids [48]. Values vary depending on the patient cohort studied, with age impacting the rate of pulmonary function loss [44,45]. Corticosteroid use may further affect pulmonary function by delaying the onset of decline [12,48]. When analyzed by genotype, pulmonary function was worse in patients amenable to exon 53 skipping compared with other mutations [12]. In our study, exon 53 skip-amenable patients receiving golodirsen showed 8.4% FVC%p loss over 3 years.

A previous study performed in an Italian and U.S. cohort of patients with DMD (*N* = 37) assessed the influence of genotypes on respiratory function, and concluded that exon 53 skip-amenable patients had the worst outcomes [12]. These patients had an annual FVC%p decline of 4.9% between the ages of 8.7 and 22.6 years, and also began with lower FVC%p at age 8.7 years compared with patients with other mutations [12] (L. Bello and E. Pegoraro, personal communication). Some of the patients in this previous study were taking corticosteroids, which have been shown to improve respiratory function, regardless of whether the steroid regimen was daily or intermittent [48].

Our study was designed to assess the safety and proof of mechanism of golodirsen, with no control group planned *a priori* for efficacy. Other limitations included the small sample size and the *post hoc* nature of the efficacy comparisons to external controls. In addition, patients in our study were evaluated for ambulation, a measure on which they were already declining at baseline. The effect of golodirsen on functions that were intact at the time of treatment initiation, such as upper limb function, needs to be explored.

Finally, our analysis of exon skipping was performed using reverse transcription PCR rather than more quantitative methods such as quantitative PCR or droplet digital PCR [49]. This method was chosen in consultation with the U.S. FDA during protocol development to demonstrate exon skipping and has been previously used to characterize exon skipping for other approved therapies [26,50].

Further quantitative analysis of golodirsen's exon-skipping properties will be an area for future research. Importantly, however, dystrophin protein expression as measured by western blot and immunofluorescence (percent dystrophin-positive fibers) was demonstrated utilizing a validated and quantitative methodology, confirming the ability of golodirsen to induce exon skipping and dystrophin protein production.

The results from this study provide evidence for the biologic activity and long-term safety of golodirsen 30 mg/kg/week in a declining population of patients with DMD and confirmed mutations amenable to exon 53 skipping. *Post hoc* comparisons of ambulation outcomes with external controls suggest functional benefit that can be measured after a 2-year period. Similarly, the cumulative 3-year decline in FVC%p among golodirsen-treated patients is compared favorably with literature-reported estimates of annual natural history decline. Overall, the data hold promise for functional benefits of golodirsen, warranting larger studies. A Phase 3, placebo-controlled, double-blind study (NCT02500381) is underway.
